# Rutin rescues oocyte developmental competence in primary ovarian insufficiency by restoring mitophagy and suppressing pyroptosis

**DOI:** 10.3389/fcell.2026.1872070

**Published:** 2026-07-15

**Authors:** Ying Zhao, Zhuo Yang, Chenyun Miao, Yuanyuan Zhang, Wenjun Xiao, Ruye Wang, Yun Chen, Ning Ren, Liuqing Yang, Haimo Qian, Heng Dai, Xiaoyi Zhu, Qin Zhang, Jing Ma

**Affiliations:** 1 Department of Chinese Medicine, The First Affiliated Hospital of Wenzhou Medical University, Wenzhou, China; 2 Hangzhou Hospital of TCM Affiliated to Zhejiang Chinese Medical University, Hangzhou, China; 3 The Third Clinical Medical College, Zhejiang Chinese Medical University, Hangzhou, China; 4 College of Life Science, Zhejiang Chinese Medical University, Hangzhou, China

**Keywords:** mitophagy, oxidative stress, primary ovarian insufficiency, pyroptosis, rutin

## Abstract

Primary ovarian insufficiency (POI) is a major cause of female infertility and endocrine dysfunction, for which effective therapies remain limited. We investigated whether rutin, a bioactive compound from traditional Chinese medicine, protects ovarian function in POI by regulating mitochondrial homeostasis and pyroptosis. *In vitro* assays using chemically injured oocytes showed that rutin alleviated mitochondrial defects, rescued developmental arrest, enhanced antioxidant signaling, promoted mitophagy, and reduced inflammatory cell death. In a cyclophosphamide-induced murine POI model, rutin restored estrous cyclicity, improved follicle development, normalized hormone levels, and enhanced ovulation, litter outcome, and *in vitro*-fertilization (IVF)-related developmental competence. Ovarian histological and molecular analyses further showed reduced inflammasome-related pyroptotic signaling, lower oxidative stress levels, and improved mitochondrial integrity. These findings identify rutin as a promising ovarian-protective candidate in POI-associated infertility and support further investigation of its therapeutic potential in female reproductive disorders.

## Graphical Abstract

**Figure:**
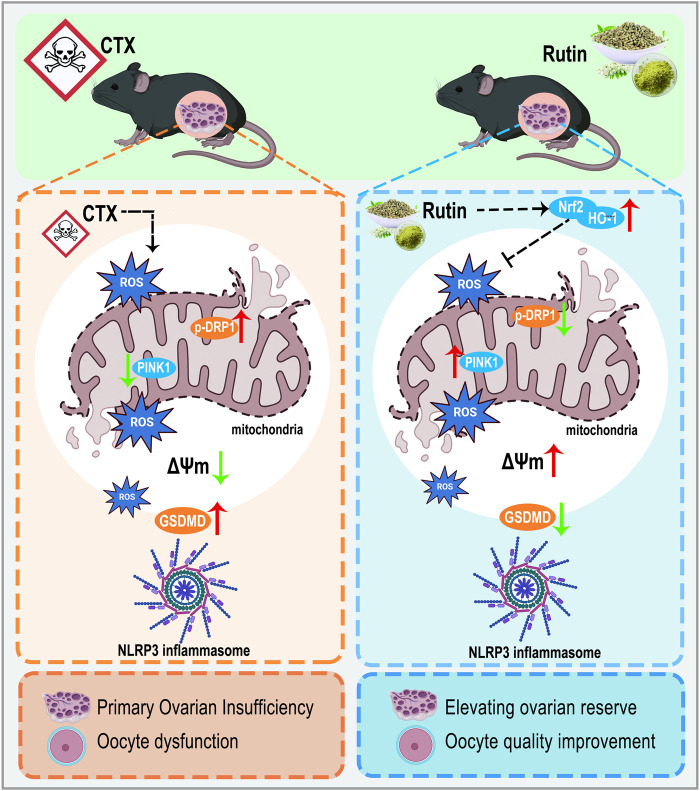

## 1 Introduction

Primary ovarian insufficiency (POI) is characterized by diminished ovarian function before the age of 40 (years) that affects 3.7% of women worldwide; it represents a significant clinical challenge and has limited effective treatment options (Heddar et al., 2022). Current consensus suggests that the complex pathogenesis of POI involves the combined effects of genetic, epigenetic, immune, and iatrogenic factors, revealing its multifaceted nature (Guo et al., 2025). However, emerging evidence highlights that mitochondrial dysfunction, oxidative stress, and NLRP3 inflammasome activation constitute the convergent pathogenic mechanisms underlying various etiologies of POI (Moustakli et al., 2025). Specifically, impaired mitophagy fails to clear the damaged mitochondria, resulting in excessive production of reactive oxygen species (ROS) and subsequent activation of the NLRP3 inflammasome; NLRP3 is a critical mediator of chronic inflammation that promotes pyroptosis in ovarian cells and accelerates follicle depletion. The interdependent relationships among mitochondrial homeostasis, oxidative stress, and inflammatory signaling create a vicious cycle that perpetuates ovarian dysfunction.

Current therapeutic approaches for POI remain suboptimal. While hormone replacement therapy (HRT) is the first-line treatment for alleviating menopausal symptoms and mitigating long-term health risks, there are concerns regarding prolonged usage and potential complications (Huang et al., 2022). The primary option for fertility restoration is oocyte donation through assisted reproductive technology, but this approach does not restore the endogenous ovarian functions (Sun et al., 2024). Experimental therapies, including stem-cell transplantation, platelet-rich plasma (PRP) therapy, and *in vitro* activation (IVA), have shown promising results in preclinical and pilot studies but face challenges related to efficacy, standardization, and biosafety, all of which preclude widespread clinical application (Ahmadi et al., 2025; Devenutto et al., 2020; Kim and Kim, 2024). This therapeutic dilemma underscores an urgent need for novel strategies capable of holistically targeting the interconnected pathogenic network of POI.

Traditional Chinese medicine (TCM), with its inherent philosophy of multitarget and multipathway regulation, offers a promising option for addressing such complex chronic diseases. Rutin, also known as quercetin-3-O-rutinoside, is a natural flavonol glycoside composed of the aglycone quercetin and the disaccharide rutinose; it is widely found in dietary and medicinal plants, including buckwheat, citrus fruits, tea, apples, berries, and several Chinese herbal medicines. Rutin has attracted attention because of its antioxidant, anti-inflammatory, antiapoptotic, and mitochondrial protective activities (Hosseinzadeh and Nassiri-Asl, 2014; Negahdari et al., 2021; Farha et al., 2022). These pharmacological properties are relevant to ovarian protection because oxidative stress and mitochondrial dysfunction are closely associated with ovarian senescence and impaired oocyte competence. The clinical importance of developing strategies that can delay ovarian senescence and preserve female fertility has also been emphasized in the context of postponing menopausal ovarian decline (Hegazy, 2020). Our previous studies identified rutin as the principal bioactive component in He’s Yangchao formula, whose capacity to enhance mitochondrial function in POI granulosa cells (GCs) and oocytes is broadly documented (Miao C. et al., 2023; Yang et al., 2024a; Yang et al., 2024b). Nevertheless, the precise mechanisms by which rutin ameliorates POI—particularly its effects on the mitophagy-NLRP3 inflammasome axis—remain incompletely elucidated. Therefore, we aimed to investigate the therapeutic mechanisms of rutin in POI in the present study. We demonstrate that rutin ameliorates cyclophosphamide (CTX)-induced POI and oocyte damage by enhancing mitophagy, improving mitochondrial function, and boosting antioxidant capacity, thereby inhibiting oxidative stress and pyroptosis, restoring ovarian function, improving oocyte quality, and enhancing fertility outcomes.

## 2 Materials and methods

### 2.1 Experimental animals and interventions

For this study, we obtained a total of 110 female C57BL/6 mice (9-weeks-old) with normal estrous cycles from the Animal Experimental Research Center of Zhejiang Chinese Medical University; the animals were acclimated for 1 week and randomly divided into five groups. Then, four groups of animals received a single intraperitoneal injection of CTX (Sigma-Aldrich; CAS: C068) at 120 mg/kg each to establish the POI model according to previously published protocols (Liu et al., 2022; Chen X et al., 2022). Model establishment was evaluated using estrous-cycle disturbance, ovarian morphology, ovarian index, follicle counts, and serum levels of anti-Müllerian hormone (AMH) and follicle-stimulating hormone (FSH). Serum estrogen (E2) level was measured as a supportive endocrine index but was not used alone as a diagnostic criterion for POI. Ten days after injection, the CTX-treated mice were evenly divided into the model, N-acetylcysteine (NAC), low-dose rutin (RutinL), and high-dose rutin (RutinH) groups. Rutin was purchased from Shanghai Yuanye Bio-Technology Co., Ltd. (Shanghai, China; CAS: B20771), while NAC (Sigma-Aldrich; CAS: A7250) was used as the positive control. The C57BL/6 mice were purchased from Shanghai SLAC Laboratory Animal Co., Ltd. (SCXK [Shanghai] 2017-0005). All animal handling procedures were approved by the Animal Experiment Welfare Ethics Committee of Zhejiang University of TCM (ethical code: IACUC-20220926-10), and the experiments were conducted in compliance with the US guidelines for the care and use of experimental animals.

### 2.2 Model and treatments

After the POI model was established using CTX at a dose of 120 mg/kg, the NAC group was given 22.75 mg/kg by gavage, while the low and high doses of rutin were administered at 75 mg/kg and 150 mg/kg by gavage, respectively, based on similar doses used in previous works (Negahdari et al., 2021; Sun et al., 2021). The model and control groups received the same volume of saline solution. The estrous cycle of each group was monitored daily via vaginal smear cytology. Briefly, vaginal lavage samples were gently collected from the vaginal opening, smeared onto glass slides, stained, and examined under a light microscope. Estrous-cycle stages were then identified according to the relative proportions of nucleated epithelial cells, cornified epithelial cells, and leukocytes; specifically, proestrus was characterized predominantly by nucleated epithelial cells, estrus by cornified epithelial cells, metestrus by a mixture of cornified epithelial cells and leukocytes, and diestrus predominantly by leukocytes with only a few epithelial cells (McLean et al., 2012; Byers et al., 2012; Cora et al., 2015). After 4 weeks of intervention, three female mice from each group were mated with 9-week-old male C57BL/6 mice, and the number of fetuses and fetal mortality rate of each group were calculated after delivery. Fifteen mice from each group received an intraperitoneal injection of 10 IU of human chorionic gonadotrophin (hCG) 48 h after injection of 10 IU of pregnant mare serum gonadotropin (PMSG) to promote ovulation. Then, cumulus–oocyte complexes (COCs) were collected within 16 h from the ampulla of the fallopian tubes after anesthesia by CO_2_ inhalation. Some of the COCs were digested with hyaluronidase to remove the granulosa cells, and the oocytes were stained for further analyses. The other COCs were placed in fertilization droplets containing an *in vitro* fertilization (IVF) medium, and the developmental potential of the fertilized eggs was observed with reference to previous oocyte maturation and fertilization studies (Soares et al., 2017; Yang et al., 2024b). For sample allocation after superovulation and oocyte retrieval, ovarian tissues from six mice per group were used for paraffin embedding and histological analyses, whereas the ovarian tissues from the remaining nine mice were reserved for Western blotting. Because mouse ovaries are small, both ovaries from a mouse were pooled to prepare a single protein sample; the final numbers of biological replicates used for Western blotting quantifications are indicated in the corresponding figure legends.

### 2.3 Germinal vesicle-stage oocyte culture and treatments

Eight-week-old female mice were injected with 10 IU of PMSG and euthanized via CO_2_ inhalation after 48 h. Then, germinal vesicle (GV)-stage oocyte isolation and *in vitro* maturation were performed with reference to established murine oocyte protocols (Randall et al., 1990; Yang et al., 2024b). In brief, the oocytes were harvested and maintained at the GV stage in M2 medium with 1 µM milrinone; the oocytes were next exposed to varying concentrations of 4-hydroperoxy cyclophosphamide (4-HC) and rutin prepared in 100 mM dimethyl sulfoxide (DMSO) stocks diluted in M2 medium containing milrinone, before culturing under mineral oil at 37 °C and 5% CO_2_. The 4-HC concentration range was designed to identify a practical injury dose for subsequent rescue experiments rather than to calculate the half-maximal inhibitory concentration (IC50). After 4 h, the oocytes were washed and transferred to fresh M2 medium. The GV breakdown (GVBD) rate was assessed at 4 h post-collection; metaphase II (MII) stage attainment and first polar body (PB1) extrusion were monitored at 16 h.

### 2.4 Network pharmacology analysis of rutin

The SwissTargetPrediction (https://www.swisstargetprediction.ch/) and HERB (http://herb.ac.cn/) websites (Daina et al., 2019; Fang et al., 2021) were used to predict the targets of rutin and its metabolites quercetin, 3,4-dihydroxytoluene, homovanillic acid, 3-hydroxyphenylacetic acid, and 3,4-dihydrophenylacetic acid (Hosseinzadeh and Nassiri-Asl, 2014). The GSE232306 dataset from the Gene Expression Omnibus (GEO) database (https://www.ncbi.nlm.nih.gov/gds/) was used to screen the differentially expressed genes (DEGs) between the POI model and normal control groups. A Venn diagram was used to identify the common DEGs between the rutin intervention and POI groups. Then, the Kyoto Encyclopedia of Genes and Genomes (KEGG) and Gene Ontology (GO) enrichment analyses of the intersected DEGs were performed using the Database for Annotation, Visualization, and Integrated Discovery (DAVID; https://david.ncifcrf.gov/) (Huang et al., 2009), and a protein–protein interaction (PPI) network was drawn using STRING (https://cn.string-db.org/) (Szklarczyk et al., 2019).

### 2.5 Enzyme-linked immunosorbent assay for detecting serum E2, FSH, and AMH levels

On the day of euthanization, a blood sample was collected from the submaxillary vein of each mouse, and the supernatant was obtained and centrifuged at 2000 *g* for 15 min to obtain the serum. The serum levels of E2 (catalog no. ELK8407, ELK Biotechnology, Wuhan, China), FSH (catalog no. ELK4808, ELK Biotechnology), and AMH (catalog no. ELK2876, ELK Biotechnology) were measured using enzyme-linked immunosorbent assay (ELISA) kits.

### 2.6 Histopathology and electron microscopy studies

The ovarian tissues were fixed in 4% paraformaldehyde after retrieving the COCs. Following tissue dehydration, paraffin embedding, and serial sectioning, the tissue slices were stained with hematoxylin and eosin (H&E) and observed under a microscope (Motic AE 2000). Follicle classification and counting were then performed according to established mouse ovarian follicle criteria (Pedersen and Peters, 1968; Myers et al., 2004). Ovarian tissues having volumes less than 1 mm × 1 mm × 1 mm were fixed with precooled glutaraldehyde and postfixed with osmium tetroxide, dehydrated, and transferred to a resin mixture, followed by staining with uranyl acetate and lead citrate. The ultrastructure of the mitochondria was observed using a transmission electron microscope (HITACHI-H7650) according to previously described ovarian ultrastructure studies (Chen et al., 2024; Miao R et al., 2023).

### 2.7 Reactive oxygen species and mitochondrial membrane potential (△Ψm) measurements of oocytes

The ROS levels in the oocytes were measured using a dichlorodihydrofluorescein diacetate (DCFH-DA) probe, and △Ψm was detected using a JC-1 probe. The oocytes were incubated with 10 μM DCFH-DA in phosphate-buffered saline (PBS) or JC-1 staining solution at 37 °C for 20 min. The fluorescence intensities were observed and images were captured under an inverted fluorescence microscope (Zeiss Axio Observer A1). The oocytes were stained using DCFH-DA (CAS: S0033S) and JC-1 (CAS: C2005) kits from Beyotime Institute of Biotechnology (Shanghai, China) according to manufacturer instructions and with reference to previous ovarian ROS and mitochondrial function studies (Chen et al., 2024; Miao C et al., 2023).

### 2.8 Immunofluorescence staining of phalloidin in ovaries

The ovarian tissue slices were fixed with formaldehyde for 20 min and washed with PBS containing 0.1% Triton X-100. Subsequently, the slices were incubated with Actin Tracker Green (phalloidin, C2201S, Beyotime Institute of Biotechnology) diluted in PBS containing 5% bovine serum albumin (BSA) and 0.1% Triton X-100 in the ratio of 1:200 at room temperature in the dark for 60 min. After washing with PBS, the slices were sealed with an anti-quenching sealing agent containing DAPI and scanned using an Olympus slide scanner (VS200, Olympus Corporation, Japan). This approach was used to visualize F-actin-associated oocyte–granulosa cell communication, as described previously (Zhang et al., 2022).

### 2.9 Immunofluorescence and confocal microscopy studies

The GV-stage oocytes were fixed in preheated 4% paraformaldehyde for 30 min and permeabilized with 0.5% triton for 20 min. The immunofluorescent oocytes were prepared using minor modifications to previous oocyte maturation and communication studies (Yang et al., 2024b; Zhang et al., 2022). After blocking with 1% BSA solution for 1 h, the oocytes were incubated with the primary antibody dilution (γ-H2AX, α-tubulin 1:1000, gasdermin D (GSDMD), NLRP3, PINK1, and HO-1 1:200) overnight at 4 °C. The next day, the oocytes were incubated with the secondary antibody at room temperature for 1 h. After staining the DNA with DAPI, the observations and photographs were recorded using an inverted fluorescence microscope or confocal laser-scanning microscope (FV 3000, Olympus). The mitochondria in the live oocytes were stained using Mito-Tracker Red CMXRos (C1035, Beyotime Institute of Biotechnology), and the nuclear DNA was counterstained using Hoechst 33342 (C1022, Beyotime Institute of Biotechnology) where applicable. The primary antibodies α-tubulin and γ-H2AX were purchased from Beyotime Institute of Biotechnology, and GSDMD (catalog no. YT7991), NLRP3 (catalog no. YT5382), PINK1 (catalog no. YN2037), and HO-1 (catalog no. YM33079) were purchased from ImmunoWay Biotechnology Company (Texas, United States).

### 2.10 Western blotting for detecting pyroptosis-associated and oxidative-stress-related protein expressions

The ovary samples were ground and lysed in RIPA lysis buffer (catalog no. ab156034, Abcam, United Kingdom) containing a cocktail of protease inhibitors (catalog no. ab65621, Abcam, United Kingdom). Western blotting was then performed by referring to previous POI studies examining oxidative stress, mitophagy, and pyroptosis (Chen Y et al., 2022; Miao R et al., 2023). After determining the protein concentration using the bicinchoninic acid assay (catalog no. P0011, Beyotime Institute of Biotechnology), the protein concentrations of all samples were adjusted to 3 mg/mL, and the proteins were denatured in a metal bath at 99 °C for 5 min. Next, SurePAGE protein electrophoresis precast gels (catalog no. M00654, GenScript, United States) were used for electrophoresis, and transmembrane electrophoresis was performed at a constant current of 400 mA for 30 min using a Rapid Transfer Buffer (catalog no. GF1816, GenScript, United States). After blocking with 5% skimmed milk, the samples were incubated with the primary antibody overnight at 4 °C, followed by incubation with the secondary antibody (catalog no. 926-32211, Licor, United States) for 1 h at room temperature on the next day. The Odyssey CLx system (Licor, United States) was used to scan the polyvinylidene fluoride membranes, and ImageJ software was used to calculate the grayscale values of the bands. The primary antibodies NLRP3 (catalog no. YT5382), GSDMD (catalog no. YT7991), caspase-1 (catalog no. YT5743), IL-1β (catalog no. YT5201), p-DRP1 (catalog no. YP1318), DRP1 (catalog no. YT1414), Mfn2 (catalog no. YT2740), OPA1 (catalog no. YN2976), HO-1 (catalog no. YM33079), Nrf2 (catalog no. YT3189), Parkin (catalog no. YT3593), and PINK1 (catalog no. YN2037) were all purchased from ImmunoWay Biotechnology Company (Texas, United States), and BAX (catalog no. 2772T) was purchased from Cell Signaling Technology (Boston, United States).

### 2.11 Statistical analysis

All statistical analyses were conducted using GraphPad Prism 8 software (GraphPad Software, Inc., United States). Unless otherwise indicated in the figure legends, the data are expressed as the mean ± standard deviation (SD) from at least three independent experiments. For normally distributed continuous outcomes involving more than two groups, statistical significance was determined by one-way analysis of variance (ANOVA) followed by Tukey’s multiple comparisons test. The litter size was analyzed using one-way ANOVA followed by Tukey’s multiple comparisons test after assessing the data distribution. The fetal mortality and live birth rates were analyzed using Fisher’s exact test based on the numbers of dead and surviving offspring. For percentage outcomes calculated from independent experimental replicates, the replicate-level percentages were compared as indicated in the corresponding figure legends. For oocyte-related scatter plots, the individual data points were displayed to show the sample size and distribution of observations. The statistical significance was set at *p* < 0.05, and the significance levels are denoted as *p < 0.05, **p < 0.01, and ***p < 0.001.

## 3 Results

### 3.1 Rutin ameliorated CTX-induced POI in mice

**FIGURE 1:**
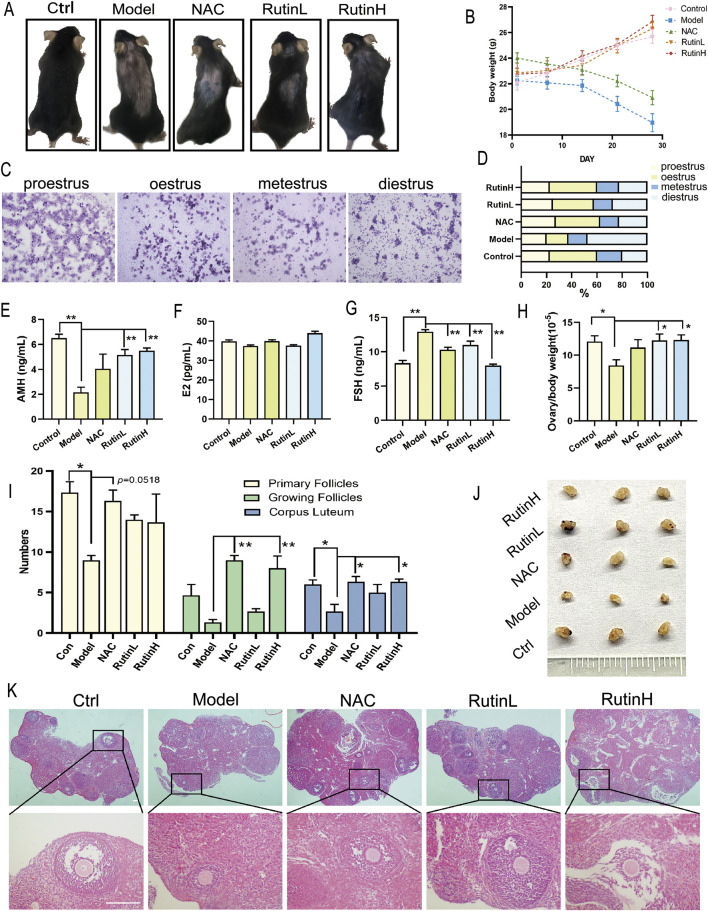
Hair, bodyweight, endocrine levels, follicle count, and ovarian index of mice with primary ovarian insufficiency (POI) after rutin intervention. **(A,B)** Hair loss and bodyweight loss among the study groups after cyclophosphamide (CTX) injection (n = 5 mice per group for bodyweight analysis). **(C,D)** Comparison of the proportions of proestrus, estrus, metestrus, and diestrus among the groups in the POI model (n = 4 mice per group). **(E–G)** Anti-Müllerian hormone (AMH), estrogen (E2), and follicle-stimulating hormone (FSH) levels measured by ELISA (n = 8 mice per group). **(H,J)** Ovarian size and ovarian index in each group, and **(I,K)** pathological observation of ovaries via hematoxylin and eosin (H&E) staining (n = 6 mice per group); scale bar = 20 μm. Data are presented as the mean ± SD. Statistical significance was determined by one-way ANOVA followed by Tukey’s multiple comparisons test. ns, not significant; *p < 0.05, **p < 0.01, ***p < 0.001.

CTX-induced POI was accompanied by weight loss and alopecia. As shown in Figure 1A, NAC and rutin prevented CTX-induced hair loss, with the high-dose rutin group showing better preventive and therapeutic efficacies. Rutin also improved weight loss compared to the model and NAC groups (Figure 1B). The four typical periods of a normal estrous cycle are shown in Figure 1C; shortened estrus and prolonged diestrus periods were observed in the POI mice, and rutin effectively improved the estrous-cycle disorder (Figure 1D). In the model group, serum AMH was significantly reduced and FSH was significantly increased (*p* < 0.01), supporting successful establishment of ovarian insufficiency together with the observed estrous-cycle disorder, ovarian atrophy, and follicular changes. Both low- and high-dose rutin increased AMH and reduced FSH levels (*p* < 0.01); the E2 levels were not significantly different among the groups (Figures 1E–G), which may be related to the PMSG-hCG-induced ovulation protocol before sample collection and may therefore be interpreted as a supportive rather than diagnostic endocrine indicator. The size and organ index of the ovary decreased significantly in the POI mice than the control group, along with the number of growing follicles and corpora lutea. High-dose rutin improved follicular growth, enhanced corpus luteum counts, and alleviated ovarian atrophy (*p* < 0.05, Figures 1H–K). Compared with the model group, no statistically significant differences were observed in the primordial follicle counts among the treatment groups (*p* > 0.05).

### 3.2 Rutin enhanced litter size and developmental potential of fertilized eggs after *in vitro* fertilization

**FIGURE 2:**
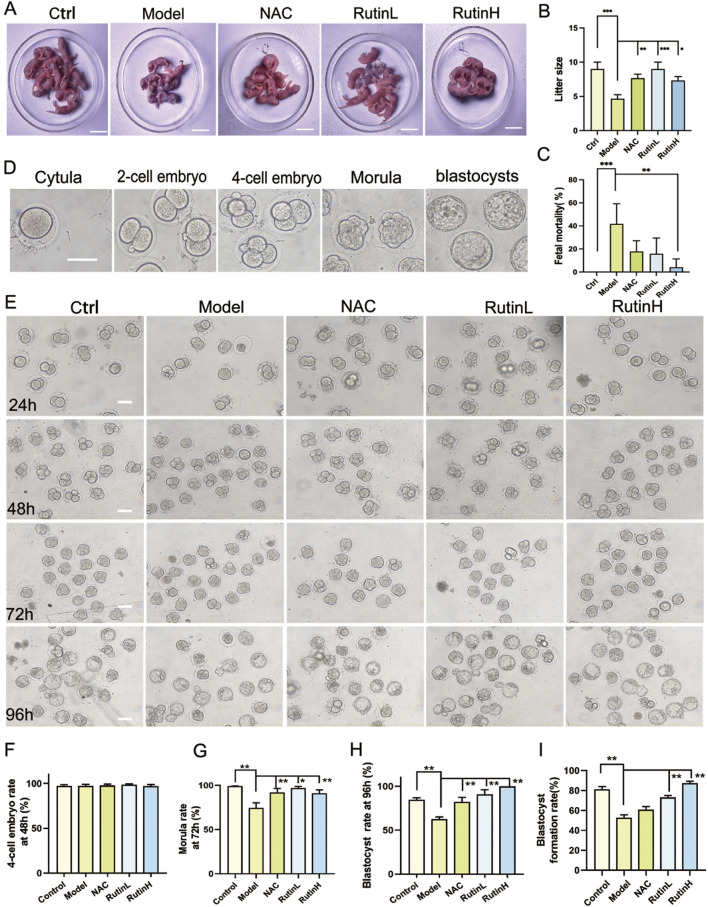
Live birth rate and developmental potential of fertilized eggs *in vitro*. **(A–C)** Changes in litter size, live birth rate, and fetal mortality after rutin intervention (n = 3 mated female mice per group). **(D,E)** Representative images showing fertilized egg development at 24 h, 48 h, 72 h, and 96 h; scale bar = 40 μm. **(F–I)** Quantification of 4-cell embryo, morula, and blastocyst formation rates at fixed time points in each group (n = 6 independent experimental replicates per group). Litter size was analyzed using one-way ANOVA followed by Tukey’s multiple comparisons test. Fetal mortality and live birth rates were analyzed using Fisher’s exact test based on the numbers of dead and surviving offspring. For **(F–I)**, percentages were calculated for each independent experimental replicate and compared using one-way ANOVA followed by Tukey’s multiple comparisons test. ns, not significant; *p < 0.05, **p < 0.01, ***p < 0.001.

As shown in Figure 2A, the litter size was significantly lower in the POI model group than the control group (*p* < 0.001); however, NAC, low-dose rutin, and high-dose rutin significantly increased the litter size compared with the model group (*p* < 0.01, *p* < 0.001, and *p* < 0.05, respectively). Live births and fetal mortality rates were analyzed as binary count outcomes. The fetal mortality rate was significantly higher in the model group than the control group (*p* < 0.001), whereas high-dose rutin significantly reduced fetal mortality compared with the model group (*p* < 0.01, Figures 2B,C). The reductions in fetal mortality in the NAC and low-dose rutin groups were not statistically significant. As shown in Figures 2D,E, the fertilized egg differentiation and development processes were observed at 24 h, 48 h, 72 h, and 96 h. The 4-cell embryo formation showed no statistical difference among the groups, while the morula and blastocyst formation rates decreased significantly in the model group than the control group. Rutin markedly increased the morula and blastocyst formation rates at 72 h and 96 h, respectively (*p* < 0.01, Figures 2D–I).

### 3.3 Network pharmacology analysis predicted the probable targets of rutin in treating POI

**FIGURE 3:**
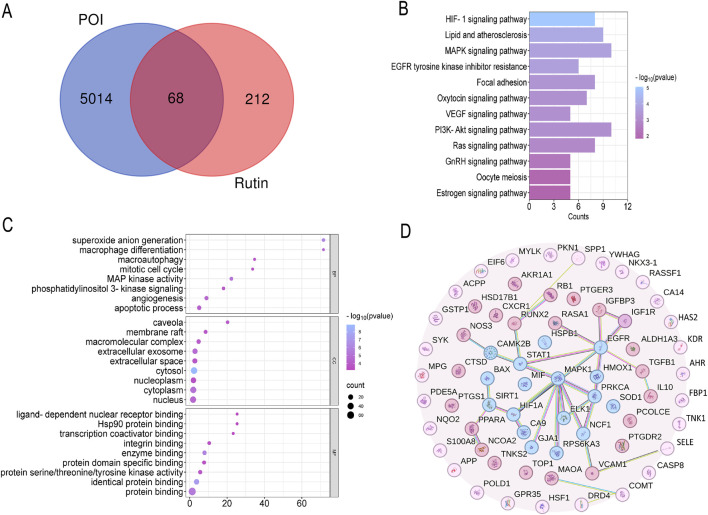
Network pharmacology analysis of rutin targeting POI. **(A)** Venn diagram depicting the intersection of differentially expressed genes (DEGs) associated with rutin and POI. **(B,C)** KEGG and GO enrichment analyses of the overlapping DEGs. **(D)** PPI network constructed with overlapping DEGs from rutin and POI using the STRING database. Statistical sample size: bioinformatics analysis based on public-database-derived targets and DEGs; no biological n value is applicable. Enrichment significance was assessed using the false discovery rate (FDR)-adjusted *p* < 0.05 threshold with Benjamini–Hochberg correction.

Based on the phenotype of rutin intervention in the POI mice, we conducted network pharmacology analysis to predict the probable targets of rutin in treating POI. Bioinformatics analyses on the GSE232306 dataset allowed us to identify 5,082 DEGs, while 280 targets of rutin were identified using the SwissTargetPrediction and HERB websites; this yielded 68 overlapping DEGs (Figure 3A). KEGG analysis showed that these genes are relevant to the estrogen and GnRH signaling pathways, oocyte meiosis, and focal adhesion, which are closely related to fertility. These genes are also connected with the MAPK, EGFR, HIF-1a, VEGF, and PI3K–AKT signaling pathways. GO analysis showed that the overlapping DEGs were related to superoxide generation, macroautophagy, mitotic cell-cycle, and apoptotic processes (Figures 3B,C). PPI network analysis showed that HMOX1, SOD1, Sirt1, and HSPB1 proteins, which are closely related to oxidative stress, as well as MAPK, EGFR, HIF1A, and STAT1 proteins, played significant roles in the treatment of POI with rutin (Figure 3D).

### 3.4 Rutin improved ovulation quantity and reduced oxidative stress in the ovary

**FIGURE 4:**
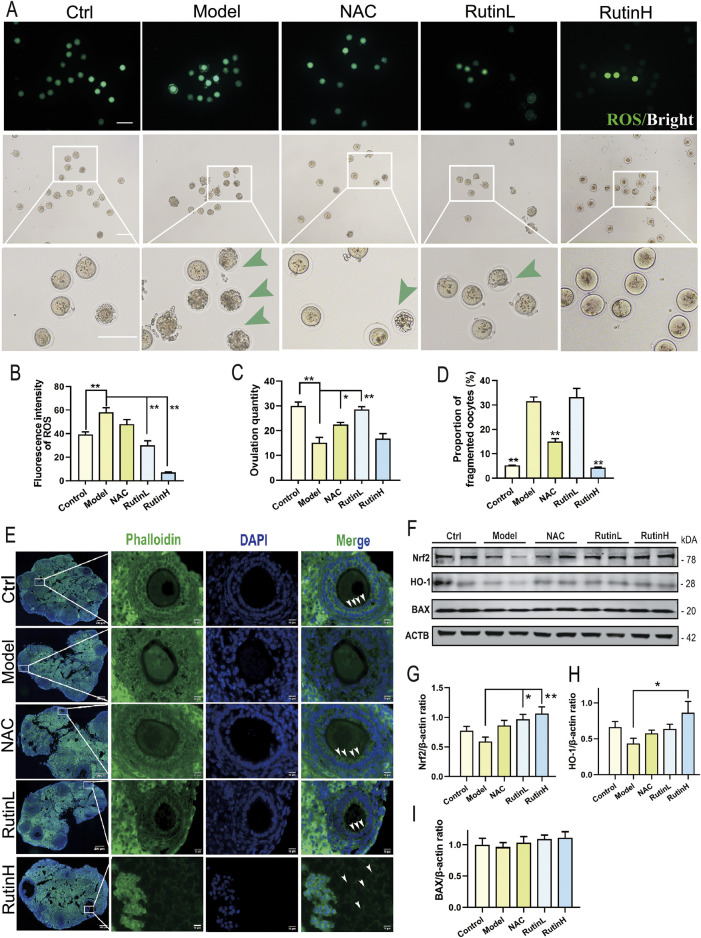
Oxidative stress alteration after rutin intervention in the POI model ovaries. **(A,B)** Reactive oxygen species (ROS) levels measured with the DCFH-DA fluorescence probe and observed under an inverted fluorescence microscope (n = 3 independent biological replicates per group); scale bar = 200 μm. **(C,D)** Ovulation quantity and proportion of fragmented oocytes (n = 6 mice per group). **(E)** Alleviation of CTX-induced oocyte-granulosa cell communication damage after rutin administration. Fibrous actin (F-actin) between the oocytes and granulosa cells (GCs) was labeled with phalloidin; white arrow: F-actin; scale bar = 10 μm. **(F–I)** HO-1, Nrf2, and BAX protein levels determined by Western blotting (n = 6 independent biological replicates per group); representative band images are shown from repeated experiments, with corresponding original Western blot images provided in Supplementary Figure S1. Data are presented as the mean ± SD. Statistical significance was determined by one-way ANOVA followed by Tukey’s multiple comparisons test. ns, not significant; *p < 0.05, **p < 0.01, ***p < 0.001.

The ovulation rate for the RutinL group was markedly higher than that for the model group, along with a decline in the proportion of fragmented oocytes (Figures 4A-D). ROS levels in both the RutinL and RutinH groups were lower than that in the POI model group (Figures 4A,B). HO-1 expression decreased in the POI mice, while high doses of rutin increased HO-1 and Nrf2 levels compared to those in the CTX-induced POI model group (*p* < 0.05, Figures 4F–H). There were no significant differences in BAX expressions between the groups (*p* > 0.05, Figure 4I).

### 3.5 Rutin restored bidirectional communication between granulosa cells and oocytes

The zona pellucida, which is located between growing oocytes and granulosa cells, maintains crosstalk between the oocytes and granulosa cells, while transzonal projections (TZPs) transport nutrients from the granulosa cells to oocytes. TZPs are rich in fibrotic actin (F-actin), which can be labeled with phalloidin. It was observed that the thin filaments in the model decreased, while both rutin and NAC restored the number of thin filaments in the TZPs and increased communication between the granulosa cells and oocytes (Figure 4E).

### 3.6 Rutin restored mitochondrial function and inhibited GSDMD-mediated pyroptosis in POI mice

**FIGURE 5:**
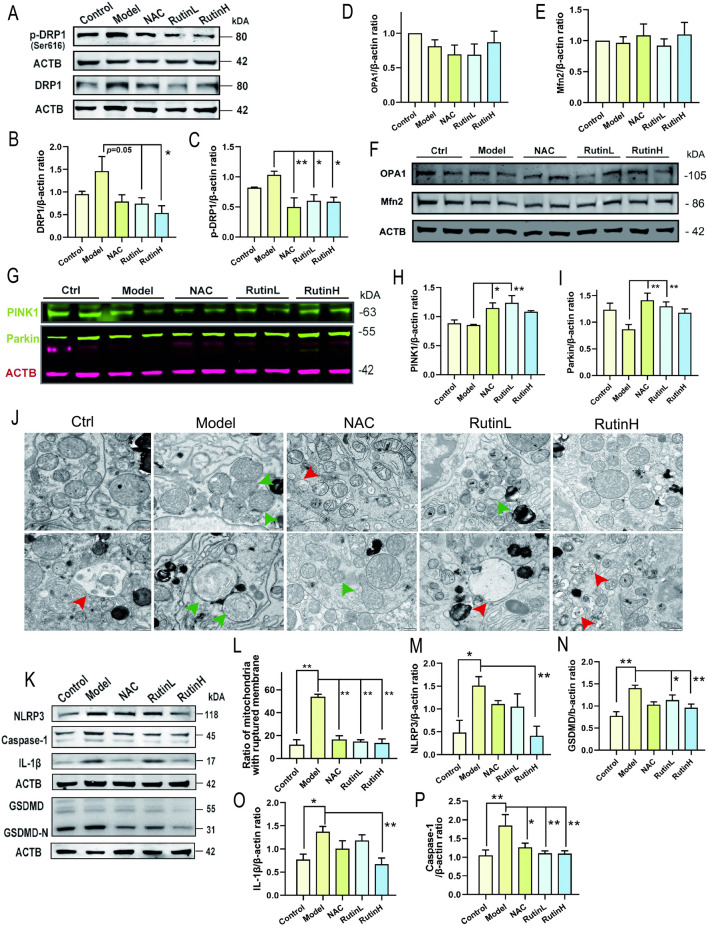
Alterations to mitochondrial functions, mitochondrial dynamics, mitophagy, and pyroptosis in response to rutin intervention in a CTX-induced model of POI. **(A–I)** Representative Western blotting images and quantitative analysis of mitophagy-associated proteins PINK1 and Parkin, mitochondrial-fusion-related proteins OPA1 and Mfn2, and mitochondrial-fission-related proteins p-DRP(Ser616) and DRP1; DRP1 was quantified from n = 4 independent biological replicates per group, and the other Western blotting bands were quantified from n = 6 independent biological replicates per group. **(J,L)** Mitochondrial ultrastructure observed by transmission electron microscopy; red arrow: autophagic bodies engulfing damaged mitochondria; green arrow: abnormal mitochondria with ruptured membrane and blurred cristae. Magnification = ×30,000; scale bar = 500 nm. **(K,M–P)** Representative Western blotting images and quantitative analysis of pyroptosis-related proteins NLRP3, GSDMD, caspase-1, and IL-1β (n = 6 independent biological replicates per group). Corresponding original Western blotting images for mitochondrial-dynamics-associated proteins and mitophagy/pyroptosis-associated proteins are provided in Supplementary Figures S2, S3, respectively. Data are presented as the mean ± SD. Statistical significance was determined by one-way ANOVA followed by Tukey’s multiple comparisons test. ns, not significant; *p < 0.05, **p < 0.01, ***p < 0.001.

As a consequence of the deficient mitophagy induced by CTX injection, we detected reductions in the expressions of PINK1 and Parkin but observed significant increases in the levels of these two mitophagy-related proteins in the rutin group than the POI model group (*p* < 0.01). The p-DRP1(Ser616) levels in the low- and high-dose rutin groups were both lower than that in the POI model group (*p* < 0.05); DRP1 expression in the high-dose rutin group was lower compared to that in the model group (*p* < 0.05), indicating alleviated mitochondrial fission. Meanwhile, Mfn2 and OPA1 expressions in each group did not show statistically significant differences, suggesting that the mitochondrial fusion process may not be altered during rutin intervention in POI (*p* > 0.05, Figures 5A–I). Observation of the mitochondrial ultrastructure using transmission electron microscopy (TEM) revealed the effects of rutin on mitochondrial functions. The mitochondrial membranes were ruptured in the POI model ovaries, and the cristae had blurred or disappeared. No autophagosomes were found in the POI ovaries. The number of abnormal mitochondria decreased, and the mitochondrial membrane maintained its integrity. Mitophagy was observed in the control and rutin groups (Figures 5J,L). The NLRP3, GSDMD, caspase-1, and IL-1β expressions increased (*p* < 0.05), and pyroptosis was activated in the CTX-induced POI model. Low-dose rutin administration decreased GSDMD and caspase-1 expressions, while high-dose rutin reduced NLRP3, GSDMD, caspase-1, and IL-1β expressions in the POI mouse model (Figures 5K,M–P).

### 3.7 4-HC exposure reduced GVBD rate and inhibited PB1 extrusion in oocytes, leading to DNA damage

**FIGURE 6:**
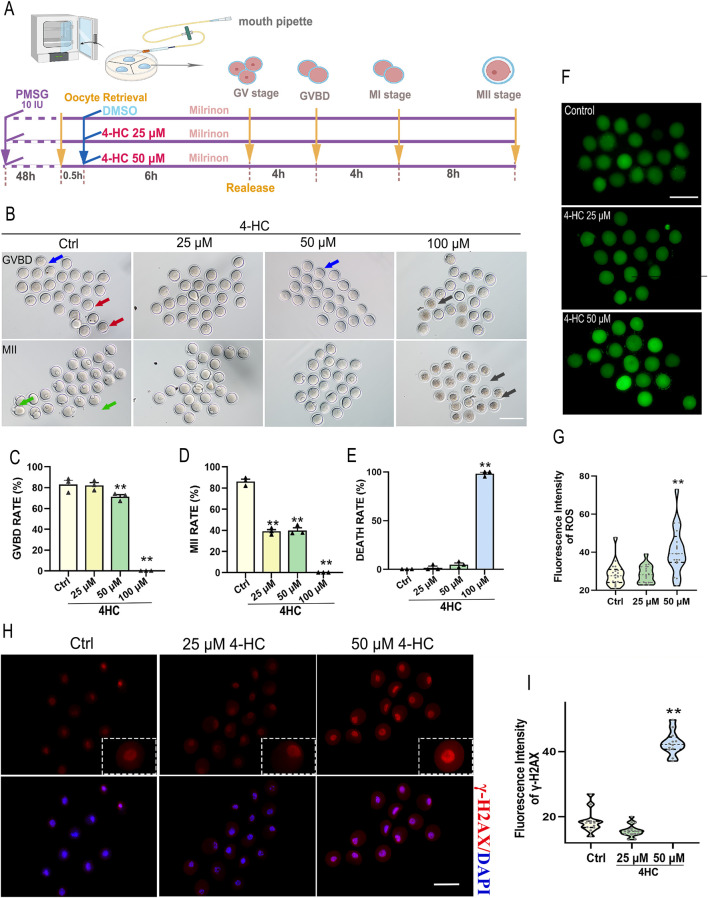
Impacts of 4-HC concentrations on oocyte maturation, oxidative stress, and DNA damage. **(A)** Experimental flowchart. **(B)** Oocyte maturation stages: blue arrows indicate germinal vesicles (GVs), red arrows indicate germinal vesicle breakdown (GVBD), green arrows indicate metaphase II (MII), and grey arrows indicate dead oocytes. **(C–E)** GVBD rate, first polar body extrusion rate, and death rate after 4-HC exposure. **(F–I)** ROS levels detected by DCFH-DA and DNA damage detected by γ-H2AX immunofluorescence; scale bar = 25 μm. For the quantitative oocyte-related panels, scatter plots show individual data points, so the sample size and distribution of the observations are presented directly in the graphs. Data are presented as the mean ± SD. Statistical significance was determined by one-way ANOVA followed by Tukey’s multiple comparisons test. *p < 0.05, **p < 0.01, ***p < 0.001.

Figure 6A outlines the *in vitro* experiment to investigate the mechanism by which 4-HC damages GV-stage oocytes. Initially, we exposed GV oocytes to varying concentrations of 4-HC for a duration of 6 h; our findings revealed that a concentration of 50 µM 4-HC significantly diminished the subsequent GVBD rate of the oocytes. Both 25 μM and 50 µM concentrations of 4-HC notably reduced the extrusion rate of the PB1 in oocytes. However, a concentration of 100 µM 4-HC resulted in a 100% mortality rate of oocytes after only 6 h of exposure (*p* < 0.01, Figures 6B–E). Using the DCFH-DA probe, we detected elevated ROS levels in the oocytes after treatment with different concentrations of 4-HC. Notably, a concentration of 50 µM 4-HC led to a significant increase in the ROS level within the oocytes (*p* < 0.01, Figures 6F,G). Further investigation through immunofluorescence staining of γ-H2AX indicated that DNA damage in the oocytes treated with 50 µM 4-HC was significantly higher compared to the control group (*p* < 0.01, Figures 6H,I). Consequently, 50 µM 4-HC was selected as the oocyte damage model in subsequent investigations because it represented the lowest concentration that produced clear oocyte injury while avoiding the complete lethality observed at 100 µM.

### 3.8 4-HC impaired mitochondrial function, reduced HO-1 expression, and increased NLRP3 expression in oocytes

**FIGURE 7:**
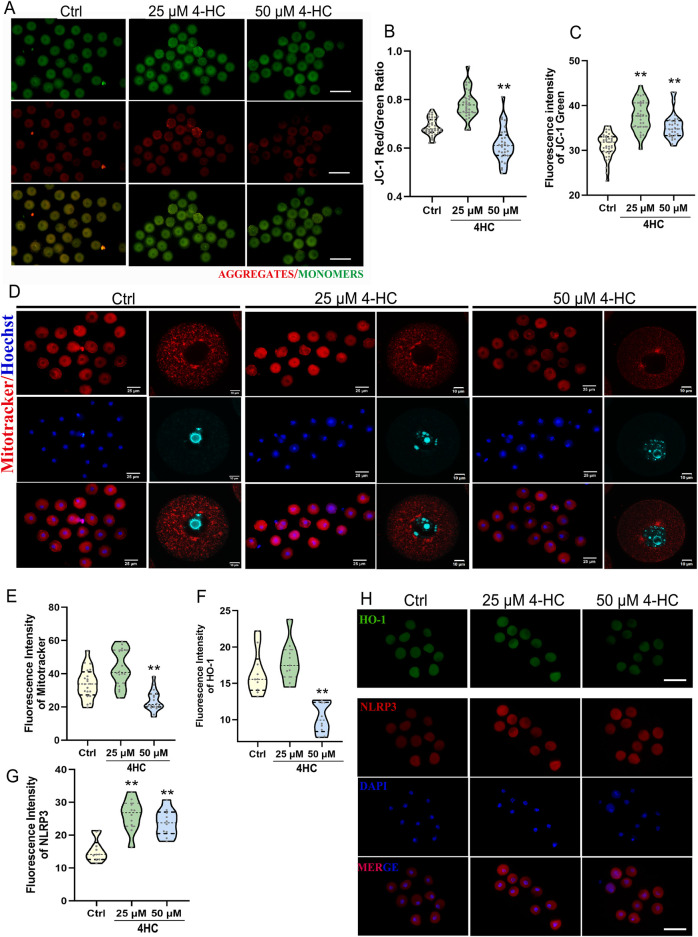
Impacts of 4-HC on oocyte mitochondrial functions, antioxidant capacity, and pyroptosis-related protein expressions. **(A–C)** Representative images and quantitative analysis of mitochondrial membrane potential in oocytes assessed using JC-1 probe; scale bar = 50 μm. **(D,E)** Mitochondrial function analysis using MitoTracker Red CMXRos. **(F–H)** Immunofluorescence staining for antioxidant protein HO-1 and pyroptosis-related protein NLRP3 in oocytes; scale bar = 50 μm. For the quantitative oocyte-related panels, scatter plots show individual data points, so the sample size and distribution of the observations are presented directly in the graphs. Data are presented as the mean ± SD. Statistical significance was determined by one-way ANOVA followed by Tukey’s multiple comparisons test. *p < 0.05, **p < 0.01, ***p < 0.001.

The mitochondrial membrane potential of the oocytes was assessed using JC-1 staining, and the oocytes were subsequently observed under an inverted fluorescence microscope. In healthy mitochondria, JC-1 aggregates and emits a pronounced red fluorescence. Conversely, when the mitochondrial membrane potential is compromised, JC-1 produces a green fluorescence. Notably, the fluorescence intensity of JC-1 green was significantly increased in both the 25 μM and 50 µM 4-HC groups. Additionally, the JC-1 red/green ratio in the 50 µM 4-HC group was significantly diminished compared to the control group, suggesting mitochondrial dysfunction induced by 4-HC (*p* < 0.01, Figures 7A–C). Using MitoTracker to label and monitor the mitochondria in the oocytes, we discovered that the fluorescence intensity of MitoTracker in oocytes treated with 50 µM 4-HC was markedly lower than that of the control group. Furthermore, oocytes in the 4-HC-induced damage group exhibited mitochondrial clustering around the nucleus under confocal laser microscopy, indicating cellular damage (*p* < 0.01, Figures 7D,E). Interventions with 25 μM and 50 µM 4-HC significantly diminished the HO-1 expressions and antioxidant capacities of the oocytes. Meanwhile, 50 µM 4-HC significantly heightened NLRP3 expression and triggered pyroptosis (*p* < 0.01, Figures 7F–H).

### 3.9 Rutin improved GVBD and MII rates after 4-HC exposure, enhanced spindle formation, boosted mitochondrial function, and reduced ROS level

**FIGURE 8:**
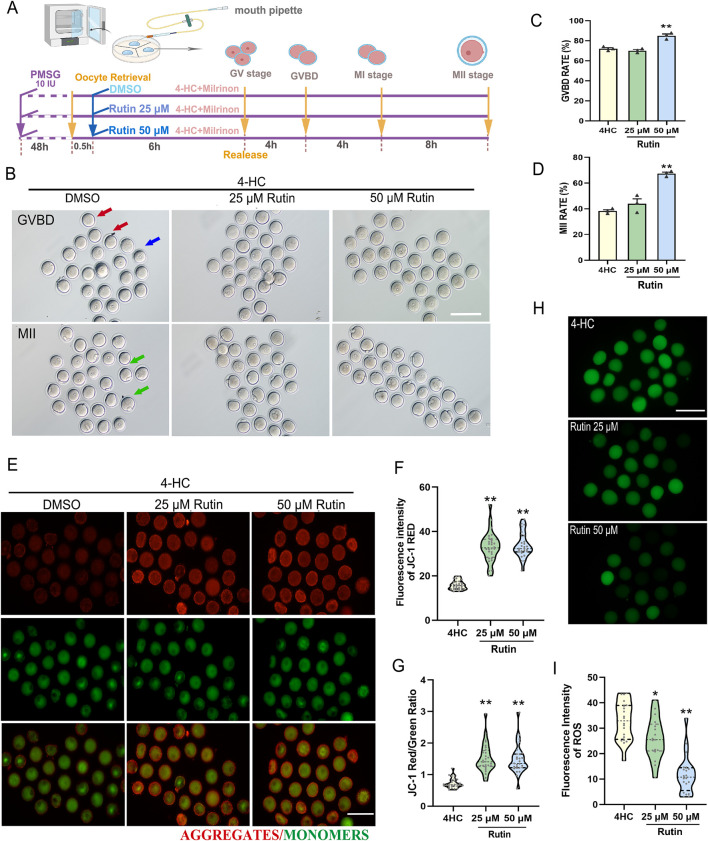
Effects of various rutin concentrations on *in vitro* oocyte maturation, oxidative stress, and mitochondrial membrane potential. **(A)** Experimental flowchart. **(B)** Oocyte maturation images; blue arrows show GV-stage oocytes, red arrows show GVBD-stage oocytes, and green arrows show MII-stage oocytes; scale bar = 50 μm. **(C,D)** GVBD rate and first polar body extrusion rate in oocytes treated with 4-HC and rutin. **(E–G)** Mitochondrial membrane potential analysis using JC-1 probe; scale bar = 50 μm. **(H,I)** ROS levels assessed with DCFH-DA probe under fluorescence microscopy; scale bar = 50 μm. For the quantitative oocyte-related panels, scatter plots show individual data points, so the sample size and distribution of the observations are presented directly in the graphs. Data are presented as the mean ± SD. Statistical significance was determined by one-way ANOVA followed by Tukey’s multiple comparisons test. *p < 0.05, **p < 0.01, ***p < 0.001.

**FIGURE 9:**
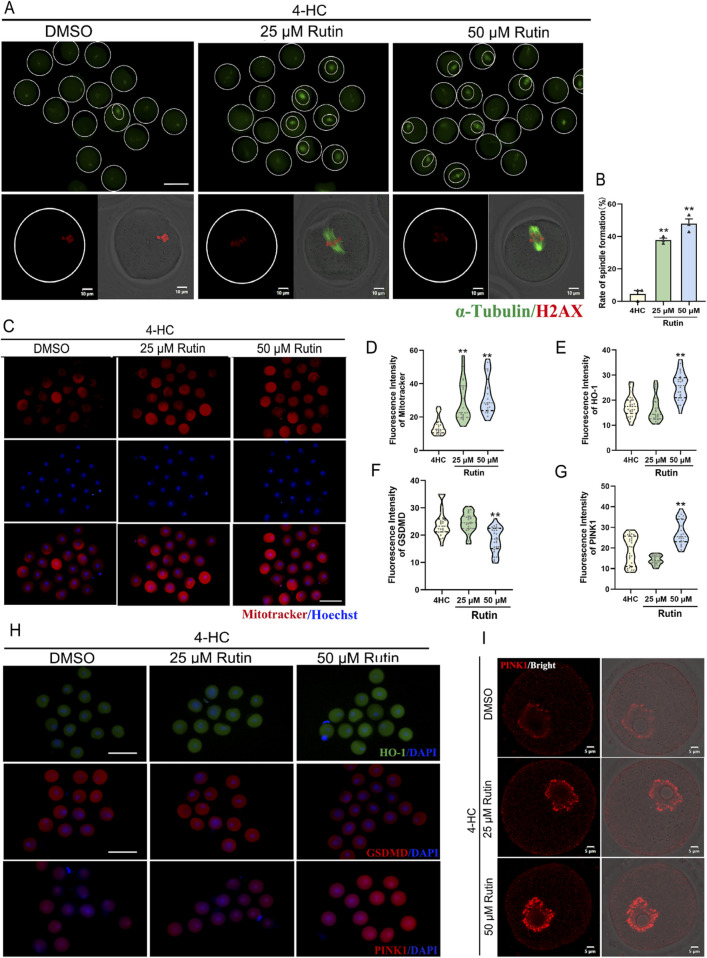
Impacts of rutin on mitochondrial functions and HO-1, GSDMD, and PINK1 expressions in oocytes. **(A,B)** Spindle formation and DNA damage analysis by α-tubulin and γ-H2AX staining under fluorescence and confocal microscopy; scale bars = 25 μm and 10 μm, respectively. **(C,D)** Representative images and quantitative analysis of the mitochondrial functions in live oocytes detected with MitoTracker Red CMXRos. **(E–I)** Representative images and quantitative analysis of HO-1, GSDMD, and PINK1 expressions in the oocytes. Nuclear DNA was counterstained with Hoechst 33342 where live-cell mitochondrial staining was performed; scale bar = 50 μm. For the quantitative oocyte-related panels, scatter plots show individual data points, so the sample size and distribution of the observations are presented directly in the graphs. Data are presented as the mean ± SD. Statistical significance was determined by one-way ANOVA followed by Tukey’s multiple comparisons test. *p < 0.05, **p < 0.01, ***p < 0.001.

Based on the oocyte dysfunction induced by 4-HC, we applied different concentrations of rutin for rescue; Figure 8A is a flowchart depicting the mechanism by which rutin rescues GV-stage oocytes. First, we found that 25 µM rutin significantly improved the MII formation rate in oocytes and that 50 µM rutin significantly reversed the reductions in GVBD and MII formation rates caused by exposure to 4-HC (*p* < 0.01, Figures 8B–D). Using the JC-1 probe, we found that rutin significantly increased the mitochondrial membrane potential and improved mitochondrial function in oocytes (*p* < 0.01, Figures 8E–G). In addition, rutin significantly reduced oxidative stress induced by 4-HC in the oocytes and decreased the ROS level (*p* < 0.01, Figures 8H,I). By culturing oocytes in M2 medium containing 4-HC and different concentrations of rutin without adding milrinone for 8 h until the MI stage, we found that rutin significantly improved spindle formation blockage caused by 4-HC (*p* < 0.01, Figures 9A,B).

### 3.10 Rutin enhanced antioxidant capacity, bolstered mitophagy and mitochondrial function, and alleviated GSDMD-mediated pyroptosis in oocytes

Upon staining with MitoTracker probes, we discovered that both 25 μM and 50 µM concentrations of rutin significantly improved mitochondrial dysfunction caused by 4-HC (*p* < 0.01, Figures 9C,D). Immunofluorescence staining of the GV-stage oocytes after intervention revealed that 50 µM rutin increased the HO-1 level in oocytes to enhance antioxidant capacity, elevated PINK1 level to activate mitochondrial autophagy, and reduced GSDMD level to inhibit the process of pyroptosis (*p* < 0.01, Figures 9E–H). By immunostaining the oocytes with PINK1 and observing mitochondrial localization under a confocal microscope, we found that 4-HC-induced damage caused the mitochondria to aggregate around the nucleus, while rutin enhanced the level of mitophagy around the nucleus (*p* < 0.01, Figure 9I).

## 4 Discussion

In the present study, we found that rutin ameliorated estrous-cycle disorder, increased AMH level, reduced FSH level, increased the organ index of the ovaries, increased the number of growing follicles in the ovary, and improved the ovulation quantity in POI mice. The data presented here indicate the superior drug efficacy of rutin in improving ovarian functions in POI mice. Subsequently, we observed the fertility of the POI model mice using both natural breeding and *in vitro* fertilization methods. Rutin increased the litter size, significantly reduced the fetal mortality rate after mating POI mice with normal male C57BL/6 mice, and markedly enhanced the morula and blastocyst formation rates at 72 h and 96 h after *in vitro* fertilization, respectively, compared with the POI model mice. These results suggest that rutin can profoundly improve oocyte quality and pregnancy outcomes in the POI model.

The PPI network results showed that HMOX1, SOD1, Sirt1, and HSPB1 proteins, which are closely related to oxidative stress, play significant roles in the treatment of POI with rutin. Furthermore, KEGG analysis showed that rutin treatment affects the estrogen and GnRH signaling pathways, oocyte meiosis, and focal adhesion, which are closely related to fertility. Rutin increased the HO-1 and total Nrf2 levels in the ovary and decreased ROS levels in the oocytes compared with the CTX-induced POI model group, indicating that Nrf2/HO-1-related antioxidant signaling may contribute to improved ovarian function. Because Nrf2 nuclear translocation was not directly examined in this study, these data should be interpreted as supportive rather than definitive evidence of Nrf2 transcriptional activation. Mitochondria serve as the primary source of cellular energy, with high demands during oocyte development and follicular growth (May-Panloup et al., 2016; Kirillova et al., 2021); in the present study, ruptured mitochondrial membranes and blurred cristae were alleviated in the rutin group, and this group also exhibited increased levels of autophagosomes upon examination of the ovarian ultrastructure via TEM. While there were no significant changes in the mitochondrial-fusion-related protein Mfn2 and OPA1 among the groups, the mitochondrial-fission-related proteins DRP1 and phosphorylated-DRP1(ser616) were significantly downregulated after rutin intervention, indicating beneficial effects on mitochondrial dynamics. These findings demonstrate that CTX administration impairs ovarian mitochondrial function, which could be restored with rutin (Chen et al., 2024; Chen X et al., 2022).

Furthermore, rutin markedly reduced the expressions of the pyroptosis-related proteins NLRP3, GSDMD, caspase-1, and IL-1β. In our *in vitro* experiment, we further discovered that rutin enhances the limited *in vitro* development of oocytes induced by 4-HC. Moreover, rutin exerts its therapeutic effects by boosting mitophagy to improve mitochondrial function, elevating HO-1 level to strengthen the antioxidant capacity and mitochondrial function, activating mitophagy, and decreasing GSDMD expression. These results confirmed the existing evidence regarding the effects of rutin on oxidative stress, mitochondrial functions, and NLRP3 inflammasomes (Chen et al., 2023a; Chen et al., 2023b; Qu et al., 2019; Wang et al., 2017; Wu et al., 2023). In addition, consistent with prior studies, we observed a correlation between the activation of GSDMD caused by NLRP3-regulated pyroptosis and destruction of mitochondrial membrane structure. Thus, we speculate that the damage to the mitochondrial membrane structure and mitochondrial function are likely due to pyroptosis, as no changes were observed in the BAX levels. Furthermore, this correlated with the phenomenon described in a recent study that GSDMD-NT first translocates to the mitochondria and causes mitochondrial damage before perforating the cell membrane; this process does not rely on the BAX proteins and mitochondrial permeability transition pores that mediate cell apoptosis (Miao C et al., 2023). Moreover, ROS are known to activate upstream NLRP3 (Billingham et al., 2022). Therefore, we conclude that rutin downregulates the activation of NLRP3 and generation of inflammasomes after improving ROS level, and this reduction of ROS and inhibition of NLRP3 inflammasomes can improve mitochondrial DNA damage and mitochondrial function. Rutin demonstrates protective effects on ovarian function and oocyte quality, eventually leading to better pregnancy outcomes. Although cleaved caspase-1 p20 was not separately detected, the coordinated changes in caspase-1, GSDMD, and IL-1β provide indirect evidence supporting altered inflammasome-associated pyroptotic signaling.

Mitophagy maintains the health of the mitochondrial network by eliminating dysfunctional mitochondria and ensuring normal metabolic activity in the ovarian cells (Iorio et al., 2021; Shi et al., 2023). As major sites of ROS production, damaged mitochondria contribute to oxidative stress when not cleared by mitophagy (Shi et al., 2023; Mishra et al., 2021). In POI mouse ovaries, impaired mitophagy likely leads to ROS accumulation, accelerating follicular atresia and ovarian dysfunction (Shi et al., 2023; Ju et al., 2025). Upon mitochondrial depolarization, PINK1 accumulates on the outer mitochondrial membrane and recruits or activates Parkin to initiate mitophagy (Vives-Bauza et al., 2010; Jin and Youle, 2012). Moderate mitophagy removes the damaged organelles to reduce mtDNA/ROS release and suppress NLRP3 activation, thereby limiting pyroptosis (Mishra et al., 2021; Ju et al., 2025). In the pathogenesis of POI, an imbalance between mitophagy and NLRP3-mediated pyroptosis may synergistically drive ovarian decline through three mechanisms: (1) insufficient mitophagy fails to effectively eliminate damaged mitochondria, leading to accumulation of ROS and mtDNA that activate NLRP3 and trigger pyroptosis; (2) excessive activation of NLRP3 induces pyroptosis that releases pro-inflammatory factors, exacerbating local ovarian inflammation and further suppressing follicular development along with mitochondrial function; (3) the vicious cycle between oxidative stress and inflammation, where ROS accumulation fuels inflammatory responses while inflammation amplifies oxidative damage and accelerates the decline of ovarian functions (Mishra et al., 2021; Shi et al., 2023; Ju et al., 2025). Our results showed that intraperitoneal CTX injection significantly downregulated PINK1/Parkin protein expressions in POI mice ovaries, whereas rutin enhanced the mitophagy levels. Our results provide valuable insights into the actions of rutin at the biochemical and molecular levels, which may aid drug discovery and development processes, ultimately contributing to the search for effective treatments against POI.

Notwithstanding the findings, this study has certain limitations. Both granulosa cells and oocytes are vital for female fertility. Hormones secreted by the granulosa cells are essential for follicular development and oocyte maturation, indicating close interactions between these two cell types, and defective communication between the oocytes and granulosa cells may lead to abnormal oocyte development (Soares et al., 2017; Zhang et al., 2022). Although we demonstrated the positive effects of rutin on the communication between granulosa cells and oocytes, we did not examine the effects of rutin on oocytes and granulosa cells *in vitro* separately. In addition, we did not directly determine Nrf2 nuclear translocation in the present study, did not separately detect cleaved caspase-1 p20, and did not use rescue or gene-intervention experiments to establish the causal order among ROS accumulation, mitochondrial dysfunction, mitophagy, NLRP3 inflammasome activation, and pyroptosis. Because rutin is a natural compound with multitarget and multipathway properties, its suppression of NLRP3-related signaling may be partly mediated by broader antioxidant or anti-inflammatory effects, such that the contributions of other inflammatory pathways such as NF-κB cannot be excluded fully. Finally, although our data indicate that rutin improves ovarian-reserve-related indices and oocyte competence in a CTX-induced POI mouse model, these findings do not establish that rutin can extend the female reproductive lifespan or postpone menopause in humans. Further investigations, including mechanistic rescue experiments, granulosa-cell-and-oocyte co-culture models, and carefully designed translational studies, are required to determine whether rutin or rutin-containing interventions may contribute to strategies aimed at delaying ovarian senescence (Hegazy, 2020).

## 5 Conclusion

In this study, we observed that rutin enhanced mitophagy-related protein expression, improved mitochondrial function, and reduced mitochondrial fission to decrease mitochondrial fragmentation, thereby boosting the antioxidant capacity, lowering the ovarian ROS level, and suppressing pyroptosis-related signaling. These effects were associated with improved endocrine indices, ovarian-reserve-related markers, litter size, live birth rate, and *in vitro* fertilization outcomes in CTX-induced POI mice. Additionally, rutin alleviates GSDMD-mediated oocyte pyroptosis by enhancing the cellular antioxidant capabilities and mitochondrial autophagy, thereby mitigating DNA damage induced by 4-HC in oocytes, improving their *in vitro* maturation competence, and increasing the formation rate of normal spindles.

## Data Availability

The raw data supporting the conclusions of this article will be made available by the authors without undue reservation.

## Glossary

4-HC: 4-Hydroperoxy cyclophosphamide
AMH: Anti-Müllerian hormone
ANOVA: Analysis of variance
COC: Cumulus–oocyte complex
CTX: Cyclophosphamide
DCFH-DA: Dichlorodihydrofluorescein diacetate
DEG: Differentially expressed gene
E2: Estrogen/estradiol
ELISA: Enzyme-linked immunosorbent assay
F-actin: Filamentous actin
FSH: Follicle-stimulating hormone
GC: Granulosa cell
GEO: Gene Expression Omnibus
GO: Gene Ontology
GSDMD: Gasdermin D
GV: Germinal vesicle
GVBD: Germinal vesicle breakdown
H&E: Hematoxylin and eosin
HO-1: Heme oxygenase-1
HRT: Hormone replacement therapy
ICSI: Intracytoplasmic sperm injection
IL-1β: Interleukin-1β
IVA: *in vitro* activation
IVF: *in vitro* fertilization
KEGG: Kyoto Encyclopedia of Genes and Genomes
MII: Metaphase II
NAC: N-acetylcysteine
NLRP3: Nucleotide-binding oligomerization domain-like receptor family pyrin domain-containing 3
Nrf2: Nuclear factor erythroid 2-related factor 2
PB1: First polar body
POI: Primary ovarian insufficiency
PPI: Protein–protein interaction
PRP: Platelet-rich plasma
ROS: Reactive oxygen species
SD: Standard deviation
TCM: Traditional Chinese medicine
TEM: Transmission electron microscopy
TZP: Transzonal projection
ΔΨm: Mitochondrial membrane potential
