# Supplementary material for: Rutin rescues oocyte developmental competence in primary ovarian insufficiency by restoring mitophagy and suppressing pyroptosis
Source: Front Cell Dev Biol. 2026 Jul 15;14:1872070. doi: 10.3389/fcell.2026.1872070 (PMC13416360; doi:10.3389/fcell.2026.1872070)

Supplementary Materials

The following supporting information is provided:

Figure S1: Original western blot images corresponding to Figure 4; Figure S2: Original western blot images corresponding to Figure 5 showing mitochondrial dynamics-associated proteins; Figure S3: Original western blot images corresponding to Figure 5 showing mitophagy- and pyroptosis-associated proteins.

Figure S1. Original Western Blot Images Corresponding to Figure 4

Original western blot images for HO-1, Nrf2, BAX, and ACTB corresponding to the quantitative results shown in Figure 4F-I. Representative blots from repeated experiments are provided. Boxes indicate the regions used for the main figure. Where applicable, membranes were stripped and reprobed as indicated.

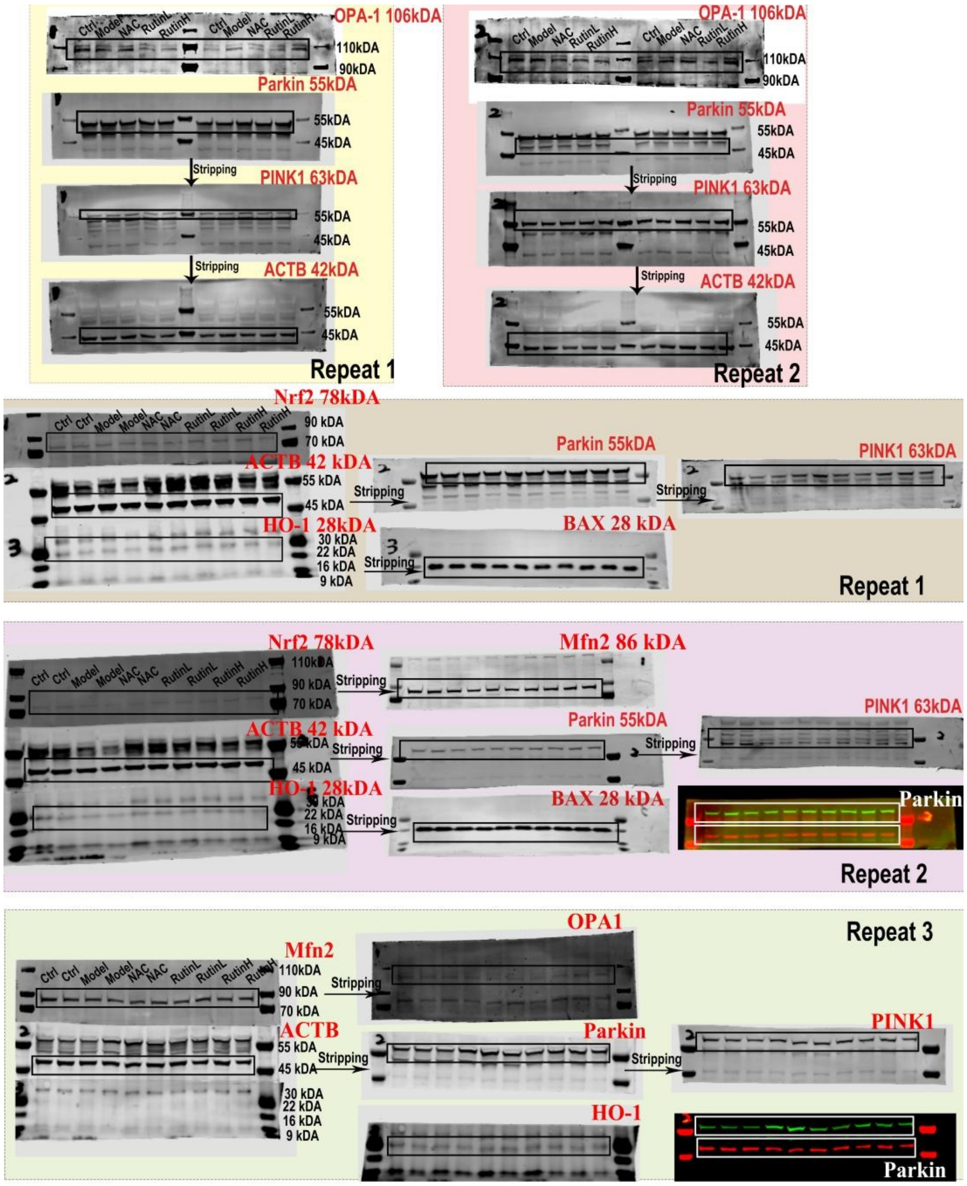

Figure S2. Original Western Blot Images Corresponding to Figure 5 Showing Mitochondrial Dynamics-Associated Proteins

Original western blot images for p-DRP1, DRP1, Mfn2, OPA1, and ACTB corresponding to the quantitative results shown in Figure 5A-I. Representative blots from repeated experiments are provided. Boxes indicate the regions used for the main figure. Stripping and reprobing steps are labeled where applicable.

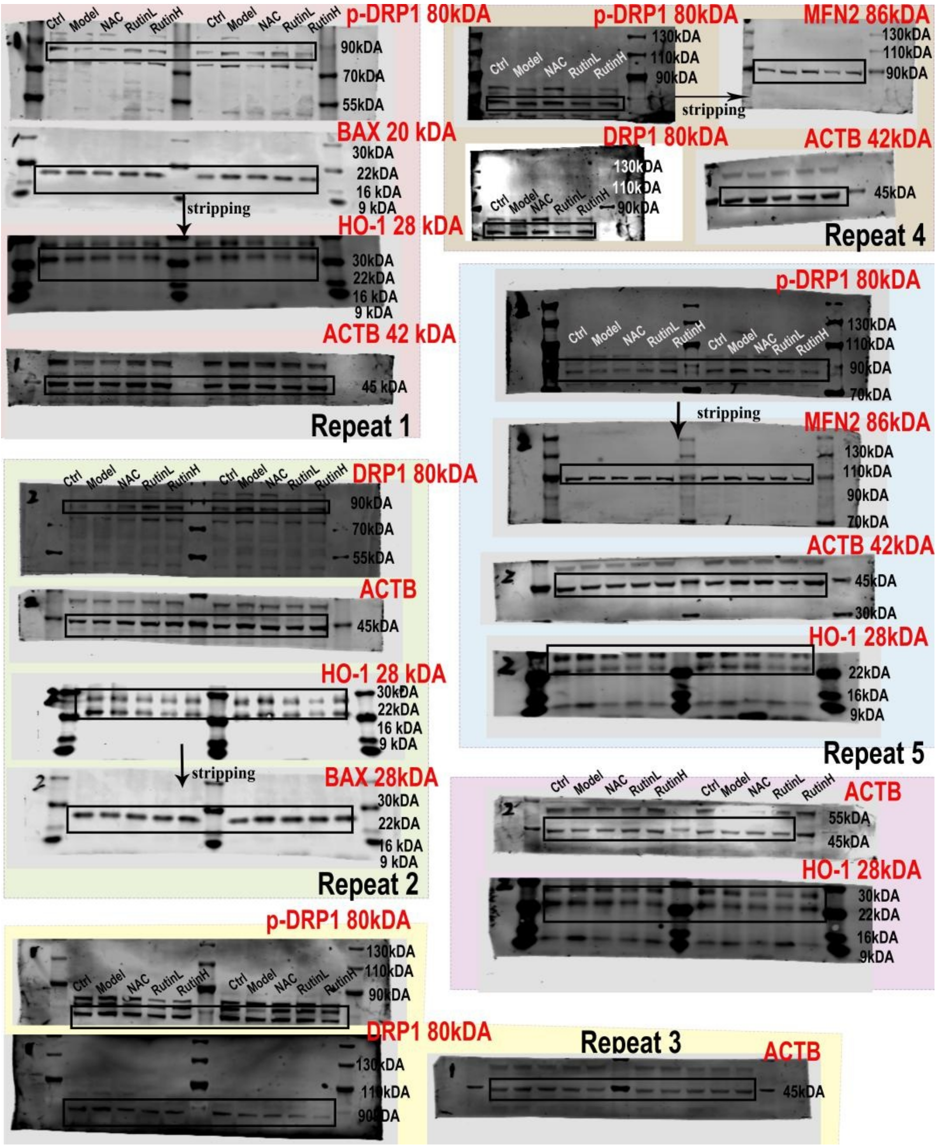

Figure S3. Original Western Blot Images Corresponding to Figure 5 Showing Mitophagy- and Pyroptosis-Associated Proteins

Original western blot images for PINK1, Parkin, NLRP3, GSDMD, caspase-1, IL-1β, HO-1, BAX, and ACTB corresponding to the quantitative results shown in Figure 5A-P. Representative blots from repeated experiments are provided. Boxes indicate the cropped regions used for the main figure. Stripping and reprobing steps are labeled where applicable.

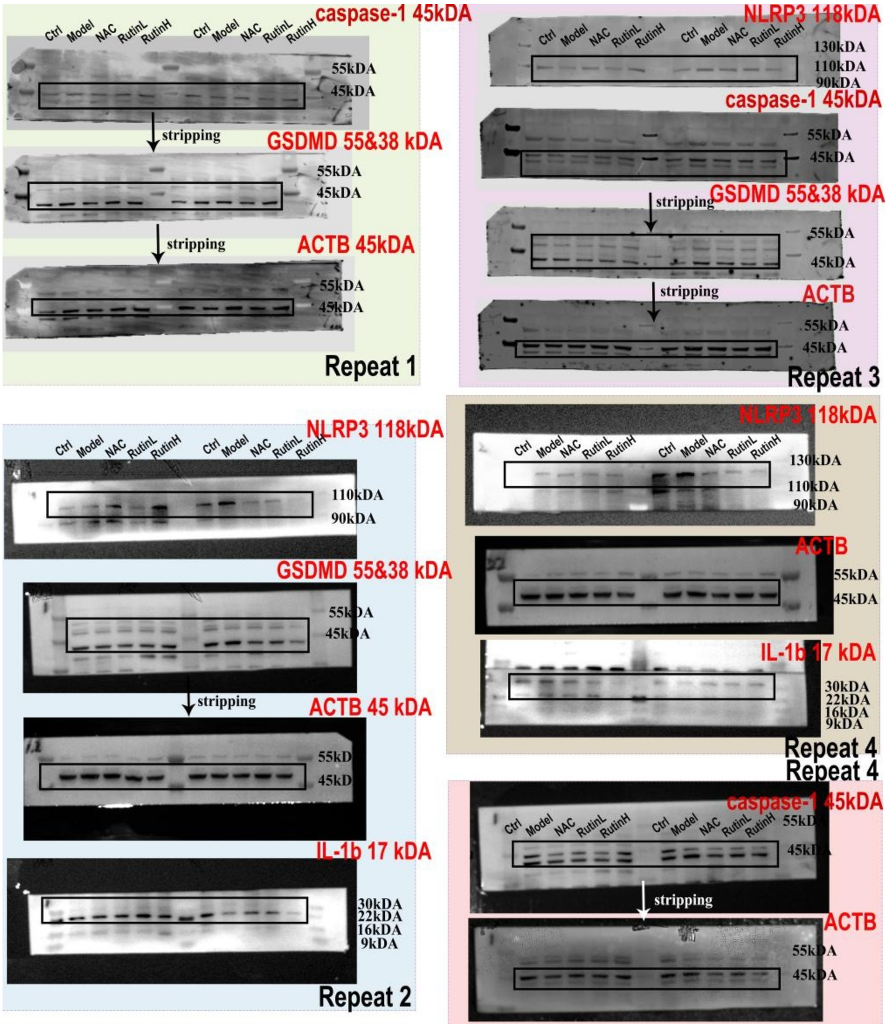

Supplement: Supplementary file 1 [file DataSheet1.pdf]
